# Anthrolysin O and fermentation products mediate the toxicity of *Bacillus anthracis* to lung epithelial cells under microaerobic conditions

**DOI:** 10.1111/j.1574-695X.2010.00740.x

**Published:** 2010-01-14

**Authors:** Taissia G Popova, Bryan Millis, Myung-Chul Chung, Charles Bailey, Serguei G Popov

**Affiliations:** National Center for Biodefense and Infectious Diseases, George Mason UniversityManassas, VA, USA

**Keywords:** pore-forming toxin, oxidative stress, anthrax, lung epithelium

## Abstract

*Bacillus anthracis* generates virulence factors such as lethal and edema toxins, capsule, and hemolytic proteins under conditions of reduced oxygenation. Here, we report on the acute cytotoxicity of culture supernatants (Sups) of six nonencapsulated *B. anthracis* strains grown till the stationary phase under static microaerobic conditions. Human small airway epithelial, umbilical vein endothelial, Caco-2, and Hep-G2 cells were found to be susceptible. Sups displayed a reduction of pH to 5.3–5.5, indicating the onset of acid anaerobic fermentation; however, low pH itself was not a major factor of toxicity. The pore-forming hemolysin, anthrolysin O (ALO), contributed to the toxicity in a concentration-dependent manner. Its effect was found to be synergistic with a metabolic product of *B. anthracis*, succinic acid. Cells exposed to Sups demonstrated cytoplasmic membrane blebbing, increased permeability, loss of ATP, mitochondrial membrane potential collapse, and arrest of cell respiration. The toxicity was reduced by inhibition of ALO by cholesterol, decomposition of reactive oxygen species, and inhibition of mitochondrial succinate dehydrogenase. Cell death appears to be caused by an acute primary membrane permeabilization by ALO, followed by a burst of reactive radicals from the mitochondria fuelled by the succinate, which is generated by bacteria in the hypoxic environment. This mechanism of metabolic toxicity is relevant to the late-stage conditions of hypoxia and acidosis found in anthrax patients and might operate at anatomical locations of the host deprived from oxygen supply.

## Introduction

*Bacillus anthracis* is a causative agent of anthrax in humans and many animal species. The inhalational form of anthrax is caused by the aerosolized spores deposited onto the surface of airways. Alveolar macrophages and dendritic cells engulf the spores and deliver them to the regional lymph nodes (Inglesby *et al.*, 1999). Some of the spores survive the bactericidal activity of phagocytes, germinate into vegetative bacteria, and subsequently become released into the lymphatic system (Guidi-Rontani, 2002; Hu *et al.*, 2006;). Further growth of bacteria leads to a hemorrhagic lymphadenitis, allowing the pathogen to break into the bloodstream and spread systemically through the circulation. Fulminant progression of infection during the bacteremic phase results in a septic shock and sudden death of the host (Inglesby *et al.*, 1999).

The toxicity of *B. anthracis* is mainly attributed to the lethal toxin (LeTx), a specific metalloprotease that inactivates mitogen-activated protein kinase kinases in the host cells. It is encoded on a plasmid XO1 along with another virulence factor, edema toxin (EdTx), which is a bacterial adenylate cyclase with an edematous activity. Both toxins, sharing a common pore-forming subunit of protective antigen, influence a number of vital cell functions, including a broad spectrum of immune responses to mitogenic and stress signals in certain cell types such as macrophages (Moayeri & Leppla, 2004). The nonimmune cell responses to the toxins include vascular and airway epithelial barrier dysfunction (Moayeri & Leppla, 2009). Some evidence indicates that in addition to LeTx and EdTx, the chromosomally located factors under certain conditions may also contribute to the *B. anthracis* virulence (Chand *et al.*, 2009).

Among these potential pathogenic factors, the *B. anthracis* hemolysin, anthrolysin O (ALO), is currently a subject of intensive investigation. It belongs to a large group of bacterial pore-forming toxins (PFTs) (Tweeten, 2005). Commonly, the interaction of PFTs with cell membranes leads to the formation of pores, thus inducing a diverse spectrum of events that depend on particular biological environment and experimental conditions (Tweeten, 2005; Ratner *et al.*, 2006;). The membrane-damaging activity of ALO has been demonstrated to interfere with innate immune mechanisms and cause cytolysis of immune cells and erythrocytes, an increase in barrier permeability of epithelial cells, escape of bacteria from the phagolysosome, induction of mitogen-activated protein kinases, as well as activation of proinflammatory response in macrophages through Toll-like receptor 4 (Klichko *et al.*, 2003; Shannon *et al.*, 2003; Park *et al.*, 2004; Popova *et al.*, 2006; Heffernan *et al.*, 2007; Bourdeau *et al.*, 2009;). However, data on the ALO properties from different laboratories are contradictory, and it seems that ALO contribution to anthrax pathogenesis depends on a variety of factors yet to be determined. For example, some authors observed no cytolytic effect of ALO even at high concentrations of the protein (Park *et al.*, 2004; Bourdeau *et al.*, 2009;). Experiments *in vivo* with ALO-null mutants revealed a substantial contribution of ALO in conjunction with phospholipases to the virulence (Heffernan *et al.*, 2007). Cowan *et al.* (2007) reported high toxicity of intravenously administered ALO, but found no protection against infection after immunization with a toxoid form of ALO. Yet, Nakouzi *et al.* (2008) demonstrated a protective effect of monoclonal antibodies against ALO in mice challenged intravenously with vegetative bacteria.

Inhalational anthrax rarely results in a primary bronchopneumonia and therefore the lung is not considered as a major site of bacterial multiplication in the earlier stages of disease. However, in the premortal stage, disseminated bacteria and secreted pathogenic products inflict strong damage to the lung (Inglesby *et al.*, 1999). Respiratory distress syndrome is the most prominent pathological feature of anthrax, which is accompanied by massive pleural effusions, edema, and hypoxia (Inglesby *et al.*, 1999). Therefore, lung epithelium has attracted attention recently as a direct target of *B. anthracis* pathogenic factors (Paddle *et al.*, 2006; Popova *et al.*, 2006; Cowan *et al.*, 2007; Lehmann *et al.*, 2009;). We have previously used human small airway lung epithelial cells (HSAECs) challenged with *B. anthracis* spores (Popova *et al.*, 2009) in order to model the exposure of lung epithelium to secreted bacterial products under conditions mimicking the late stage of infection in the hypoxic lung. In this model, bacteria grew on epithelial cells in an atmosphere of 5% carbon dioxide under static conditions known to create a hypoxic (microaerobic) environment of limited oxygen availability due to the consumption of oxygen by growing epithelial cells (Metzen *et al.*, 1995; Pettersen *et al.*, 2005;) and bacteria (Hassett, 1996; Wyckoff *et al.*, 2002; Ju *et al.*, 2005;). We found that during the exponential phase of bacterial growth, HSAECs remained viable and responded to infection through several signaling pathways, including the PI3K/AKT controlling the integrity of epithelium (Popova *et al.*, 2009). Further experiments revealed that bacteria generated highly toxic soluble factors during the stationary phase of growth. Unexpectedly, the toxicity did not correlate with the production of LeTx and EdTx by bacteria. Cells of other types were also found to be susceptible. Such a dominant cytotoxic effect of *B. anthracis* independent of LeTx and EdTx has not been described previously. We demonstrate that this results from the generation of reactive oxygen species (ROS) by the mitochondria in the host cells exposed to the secreted bacterial products under conditions resembling hypoxia and acidosis typically found in terminal anthrax patients. Among these products, succinic acid (SA) is formed as a result of anaerobic fermentation and acts synergistically with ALO. The contribution of ALO to the toxicity of Sups depends on the *B. anthracis* strain. In support of this mechanism, we show that the cells can be partially protected from death by inhibiting ALO with cholesterol, increasing the pH of Sups, or by catalytic decomposition of ROS and downstream toxic products such as hydrogen peroxide (H_2_O_2_) and peroxynitrite.

## Materials and methods

### Materials

All reagents were from Sigma-Aldrich, unless specified otherwise. The fluorescent dyes were purchased from Molecular Probes. All cell culture reagents and formulated media were from Mediatech Inc. (VA). Complete Serum-Free Medium® (CSFM) is a proprietary serum-free and low-protein formulation based on DMEM/F-12 and 5% of RPMI 1640, and McCoy's 5A. It does not contain any insulin, transferrin, cholesterol, growth, or attachment factors, and contains trace elements, high-molecular-weight carbohydrates, extra vitamins, a nonanimal protein source, and a high-quality bovine serum albumin (BSA) (1 g L^−1^). All culture media were certified as lipopolysaccharide free. Cholesterol was dissolved in ethanol and then further diluted in the culture medium or used as a water-soluble complex (CD-cholesterol), which contained 40 mg of cholesterol per gram of methyl β-cyclodextrin (MbCD). Halt proteinase inhibitor cocktail (1 ×) from Pierce (IL), contained pepstatin A, E64 benzamidine, leupeptine, PMSF, and bestatin, and was supplemented with 200 μM phosporamidon and 1 mM phenanthroline.

### Recombinant ALO

The protein was expressed, purified, and characterized as described previously (Klichko *et al.*, 2003). Briefly, the His-6-tagged protein was expressed in *Escherichia coli*, isolated using a Ni-chelate column, and stored at −20 °C in 50% glycerol, 50 mM Tris-HCl (pH 7.5). In sodium dodecyl sulfate polyacrylamide gel electrophoresis, freshly isolated protein displayed a single band with a molecular weight of around 60 kDa corresponding to monomeric ALO. In the purification stage, all solutions were prepared using lipopolysaccharide-free water and disposable plastic ware. In the experiments with HSAECs at the highest ALO concentration used (50 ng mL^−1^), the lipopolysaccharide content was below 20 pg mL^−1^. Control experiments showed no lipopolysaccharide-related toxicity in the Alamar Blue test when lipopolysaccharide was incubated with HSAECs at a concentration of 10 ng mL^−1^ (500-fold higher than in the experiments with ALO) in the presence or absence of SA for 2 h (Supporting Information, Fig. S1).

### Bacterial strains and preparation of spores and culture supernatants (Sups)

The Sterne 34F2 strain (pXO1^+^, pXO2^−^) was obtained from the Colorado Serum Co. (Boulder, CO). The generation and characterization of the plasmid-less delta-Sterne derivative of 34F2 strain and the preparation of spores have been described elsewhere (Bradburne *et al.*, 2008). We also refer to these strains as Sterne and dSterne, respectively. The Sterne 7702 strain (pXO1^+^, pXO2^−^) and the hemolysins' deletion mutants have been reported previously (Heffernan *et al.*, 2007). The mutant phenotypes were BDT101 (7702, Δ*aloA::km*^*r*^); BJH250 (7702, Δ*plcBsmcA::sp*^*r*^Δ*plcA::em*^*r*^); and BJH258 (7702, Δ*aloA::km*^*r*^*;*Δ*plcBsmcA::sp*^*r*^Δ*plcA::em*^*r*^). Spores were prepared using lipopolysaccharide-free water in the final washing steps. Bacterial culture supernatants (Sups) were produced by inoculation of spores (final 6 × 10^6^ spores mL^−1^) into the lipopolysaccharide-free CSFM (4 mL per well; liquid depth of 10 mm) and incubation at 37 °C, 5% CO_2_ in 12-well tissue culture plates for 24 h under static conditions. For growth curve measurements, the content of the well was carefully mixed by pipetting and the turbidity of the bacterial suspension was recorded at 600 nm using a microplate reader. For aeration, the cultures grown under the above conditions were additionally shaken at 200 r.p.m. Bacteria were removed by 2 × centrifugation at 3000 ***g*** for 30 min at 4 °C and the Sups were supplemented with antibiotics (100 U mL^−1^ of streptomycin and 100 μg mL^−1^ of penicillin) to exclude the potential effect of bacterial contamination. The Sups were used within several hours after cultivation.

### Cultivation of cells and challenge experiments

HSAECs and HUVECs (human umbilical vein enodothelial cells) were from Cambrex Inc. (MD); Caco-2 and Hep-G2 cells were from ATCC (Manassas, VA). Cells were seeded at a density of 2.5 × 10^4^ per well and grown till confluence in Ham's F-12 medium supplemented with nonessential amino acids, pyruvate, β-mercaptoethanol, and 10% fetal calf serum at 37 °C, 5% CO_2_ using 96-well culture plates. In challenge experiments, the growth medium was removed, the cells were washed three times with warm HEPES-buffered saline (HBSS), and then incubated at 37 °C, 5% CO_2_ without shaking with 200 μL per well of Sups, unless specified otherwise.

The viability of the cells was determined routinely using the redox dye Alamar Blue after exposure to Sups for 2 h, unless specified otherwise. Alamar Blue is a water-soluble, nontoxic, fluorometric/colorimetric growth indicator. Cellular growth and metabolism reduce the dye and cause a change in color to red. Briefly, the cells were washed with HBSS, 100 μL of Alamar Blue in CSFM was added, incubated with cells until the accumulation of sufficient signal (typically from 30 min to 1 h), and fluorescence was measured at 530/584 nm. The metabolic activity of the treated cells was calculated relative to the mock controls.

### Assay kits

The following assay kits were used according to the manufacturers' protocols: the Cytotoxicity Detection Kit for lactate dehydrogenase (LDH) from Roche Diagnostics; ATPLite-M for ATP from Packard BioScience Company; Nitrate/Nitrite Colorimetric Assay Kit from Cayman Chemical; and the lipopolysaccharide E-Toxate test from Sigma. The Megazyme kit for SA was from International Ireland. The assay allows a direct analysis of SA in complex biological products and foods. Its specificity is based on the conversion of succinate to succinyl-CoA by succinyl-CoA synthase. This enzyme also reacts with itaconic acid. However, *Bacillus* species are not known to produce itaconic acid (Martin *et al.*, 1961), which is therefore unlikely to interfere with the analytical results. The assay calibration curves were obtained with SA dissolved in water, CSFM, or spiked into Sterne 34F2 Sup (Fig. S2). The results demonstrated the low influence of the Sup components other than SA on the assay.

### Dot-blot analysis of ALO in Sups

Cholesterol was dissolved in ethanol at a concentration of 300 μg mL^−1^ and 2 μL of solution was dot-blotted onto the 2 × 2 cm PVDF membranes. The membranes were air dried, blocked with 5% of dry milk in phosphate-buffered saline (PBS) for 1 h at room temperature, and incubated with 10 mL of Sups, known amounts of recombinant ALO in 10 mL of CSFM, or CSFM at 4 °C overnight. After 2 × wash with PBS/0.1% Tween 100 for 1 h, the membranes were incubated with α-streptolysin O rabbit antibody (Capricorn Products, 1 : 1000 in the wash buffer) for 2 h at room temperature, 2 × washed for 30 min, and finally incubated for 1 h with a secondary HRP-conjugated goat α-rabbit antibody (Sigma-Aldrich, 1 : 10 000). The membranes were developed using the SuperSignal West Femto Maximum Sensitivity Substrate (Pierce), and band intensities were measured using a Molecular Imager ChemiDoc XRS System (Bio-Rad). The intensities of the bands were calculated relative to untreated controls after densitometry using the quantity one 4.6.5 program (Bio-Rad). All measurements were performed in triplicate.

### Microscopy

Nikon's TE-2000 inverted microscope along with a Nikon 90i upright were used in combination with a Nikon C1 point scanning confocal and Photometrics ES2 cooled monochrome CCD to record both live cell and fixed preparations. nis elements software was used for both the acquisition and the analysis of the resulting images and datasets.

### Statistical analysis

All measurements were performed in triplicate, and all experiments were repeated at least twice, with consistent results. Error bars in all the figures indicate 95% confidence intervals in a two-tailed *t*-test.

## Results

### *Bacillus anthracis* grown under microaerobic conditions secretes highly toxic products distinct from LeTx and EdTx

**Fig. 1:**
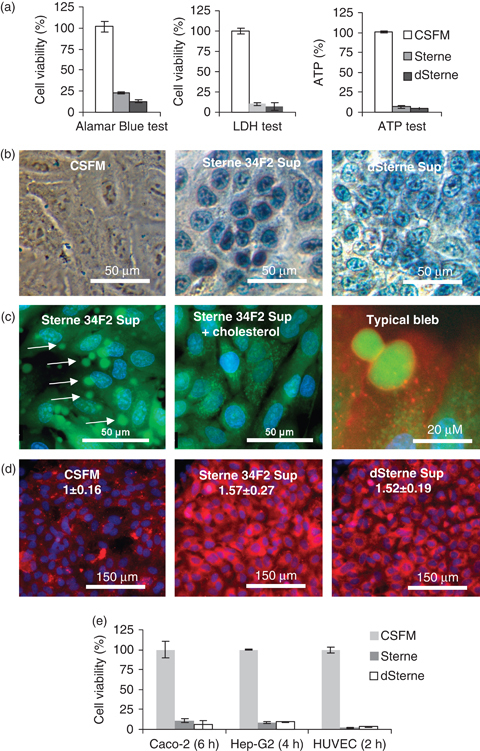
*Bacillus anthracis* generates toxic secreted products in static cultures. (a, b) Bacteria were grown in the CSFM. The viability of HSAECs relative to untreated controls was tested after incubation with Sups for 2 h at 37°C, 5% CO_2_ using the Alamar Blue test (a, left panel), LDH test (a, middle panel), intracellular ATP test (a, right panel), and the Trypan Blue permeability test (b). (c) Formation of membrane blebs in HSAECs incubated with Sterne 34F2 Sup. HSAECs were loaded with Calcein AM (5 μM, green), Hoechst 33342 (2.5 μg mL^−1^, blue), and wheat germ agglutinin conjugated with Alexa Fluor 555 (5 μg mL^−1^, red), washed with HBSS, and treated with the Sterne Sup for 30 min. The cells demonstrate a transient (up to 30-min postexposure) appearance of numerous membrane blebs (left panel, arrows). The addition of CD-cholesterol (1 μg mL^−1^) to the Sup for 30 min completely prevents blebbing (middle panel). A typical bleb (right panel) contains a part of the cell cytoplasm (green) surrounded by the cytoplasmic membrane (red). Similar results were obtained with dSterne Sup (not shown). (d) HSAECs grown on slides were incubated with Sups for 2 h at 37°C, 5% CO_2_, stained with Annexin V-conjugated Cyt3.18 dye (red) and nuclear DAPI dye (blue) for 10 min, fixed, and mounted for imaging. Numbers show the mean intensity of fluorescence (± 95% confidence interval) inred channel relative to the control cells incubated in CSFM. The intensity was evaluated with nis elements software (Nikon) using 10 independent fields of view in three different culture wells for each experimental condition. (e) Viability of Caco-2, HEP-G2, and HUVEC cells in the Alamar Blue test relative to the untreated control cell after the indicated times of exposure to Sups at 37°C, 5% CO_2_. Error bars represent 95% confidence interval of mean.

In our previous report, we used an experimental system in which cultured HSAECs were infected with *B. anthracis* spores and the responses of the cells to the germinating spores and vegetative bacteria were studied (Popova *et al.*, 2009). The results indicated that during the first 8 h of incubation, bacteria of the toxigenic Sterne strain 34F2 growing in the culture medium on top of the confluent HSAECs under static conditions at 37 °C and 5% CO_2_ did not influence HSAEC viability, although during this period of time, bacteria secreted more than 3 μg mL^−1^ of LeTx and EdTx. In the current study, we found that longer incubation times resulted in a progressive loss of HSAEC viability and wanted to elucidate the nature of this toxic effect. Analysis of the bacterial cultures grown in the absence of HSAECs revealed that the toxicity resided in the supernatant fraction (Sup). The results in Fig. 1a show that Sups of the 24-h cultures grown under the conditions of the experiments with HSAECs demonstrated a high level of toxicity when HSAECs were exposed to Sups for 2 h. The pelleted and washed bacteria resuspended in the original volume of fresh culture medium had no toxicity (not shown), indicating that toxicity was due to the activity of secreted bacterial factors.

Unexpectedly, the Sup of dSterne strain, the nontoxigenic plasmidless derivative of Sterne 34F2, showed similar potency of killing HSAEC as the Sup of the toxigenic Sterne 34F2. In the case of both the strains, HSAECs treated with Sups displayed a strong decrease in metabolic activity in the test with Alamar Blue (Fig. 1a, left panel), a depletion of intracellular LDH (Fig. 1a, central panel) and ATP (Fig. 1a, right panel). Cytoplasmic membrane perturbations were evident in the increased cell permeability to Trypan Blue (Fig. 1b) and the transient formation of membrane blebs (Fackler & Grosse, 2008) visualized with Calcein AM (Fig. 1c, left and right panels). This dye generates green fluorescent Calcein upon cleavage in the cell cytoplasm. The addition of CD-cholesterol to the Sups prevented bleb formation completely (Fig. 1c, middle panel). Staining with Annexin V-conjugated Cyt3.18 dye revealed strong fluorescence of Sup-treated cells, in comparison with untreated cells, indicating increased accessibility of the dye to the membrane phosphatidylserine normally located in the inner leaflet of the cell membrane. The toxicity was not specific to HSAECs as other cell types such as HUVECs, Caco-2, and Hep-G2 cells were also found to be highly susceptible (Fig. 1e).

**Fig. 2:**
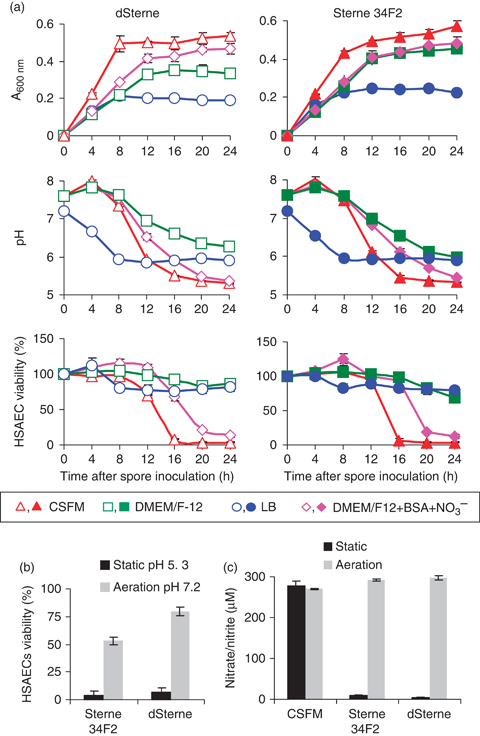
The microaerobic growth of *Bacillus anthracis* under different culture conditions. (a) Static cultures grown at 37°C, 5% CO_2_ in the nitrate-rich CSFM (triangles), Luria broth (circles), DMEM/F-12 (squares), and DMEM/F-12 supplemented with 300 μM NaNO_3_ and 1 μg mL^−1^ of BSA (diamonds) generate acidic and toxic Sups. Open symbols, dSterne; closed symbols, Sterne 34F2. (b and c) Aeration by shaking at 200 r.p.m. of the *B. anthracis* cultures grown at 37°C, 5% CO_2_ in CSFM reduces the toxicity and acidification of Sups (b) and abrogates the consumption of nitrate from the medium (c). The final pH or the cultures is shown in (b). Error bars represent 95% confidence interval of mean.

Comparison of Sups generated in different media showed that the highest toxicity was detected in the case of bacteria grown in the CSFM medium, in contrast to DMEM/F-12 and Luria broth (Fig. 2a, lower panels). We noticed that CSFM contains a high amount of nitrate (∼300 μM in CSFM vs. <1 μM in DMEM/F-12) and suggested that it may play a role in toxicity through changes in bacterial metabolism. Nitrate is known to replace oxygen for bacterial respiration under microaerobic (reduced oxygen) conditions present in static bacterial cultures (Hassett, 1996; Wyckoff *et al.*, 2002; Ju *et al.*, 2005; Pettersen *et al.*, 2005;). The resulting process of denitrification (Ju *et al.*, 2005; Rock *et al.*, 2005;) can lead to the consumption of nitrate. Indeed, microaerobic cultivation of *B. anthracis* in CSFM under static conditions was accompanied by a reduction in the nitrate content (Fig. 2b) and acidification of the medium to pH 5.3–5.5 (Fig. 2a, middle panels), indicating anaerobic acid fermentation.

Complementation of DMEM/F-12 with nitrate increased toxicity (Fig. 2a, lower panes), while aeration of the cultures by shaking prevented the consumption of nitrate (Fig. 2b) and reduced the toxicity and acidity of Sups (Fig. 2c). Bacterial growth correlated with a reduction in the pH of the Sups, but no acute toxicity was observed until the late stationary growth phase with pH<6 (Fig. 2a). Overall, the observed effects were consistent with the onset of metabolic acidosis as a result of anaerobic fermentation under the conditions of bacterial respiration on nitrate induced by low oxygen availability in static cultures.

### Microaerobic Sups display cholesterol- and pH-dependent components of toxicity

**Fig. 3:**
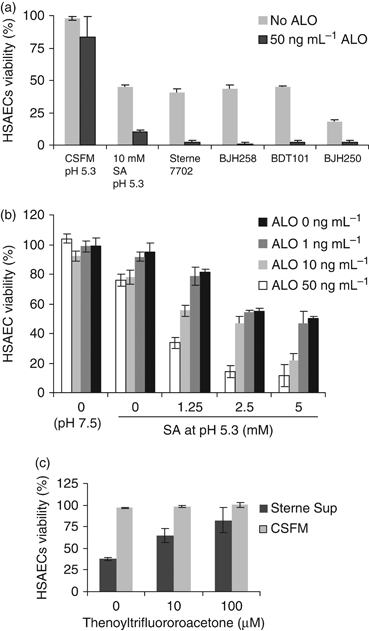
Synergistic effect of ALO and SA on HSAEC viability. (a) Recombinant ALO (50 ng mL^−1^) was spiked into Sups of the hemolysin mutants strains, Sup of the parental strain Sterne 7702, and the solution of SA in CSFM titrated to pH 5.3 with HCl. The mixtures were applied to HSAECs for 2 h. (b) Different concentrations of SA in CSFM were titrated with HCl to pH 5.3, mixed with ALO, and applied to HSAECs for 2 h. Cell viability after treatment is shown relative to incubation in CSFM (pH 7.5). (c) HSAECs were treated with dSterne Sup (gray bars) or incubated in control CSFM (open bars) in the presence of indicated concentrations of thenoyltrifluoroacetone for 30 min. (a–c) The viability of the cells was tested with Alamar Blue as described in Materials and methods. Error bars represent 95% confidence interval of mean.

In order to determine whether the reduction of pH observed during bacterial growth was required for toxicity, we tested the activity of neutralized Sups. Consistent with the results shown in Fig. 2, no toxicity to HSAECs was found at pH>6.0 when Sups were titrated with NaOH or diluted with a neutral culture medium (not shown). However, a concentration-dependent reduction of toxicity was detected when Sups were diluted using CSFM titrated with HCl to pH 5.3, corresponding to the acidity of Sups (Fig. S3). In the control experiments, the titrated medium did not show a substantial toxicity, indicating that it was associated with the pH-dependent activity of secreted bacterial product(s), but not the pH itself. *Bacillus anthracis* is known to generate a number of fermentation products, including formic, acetic, lactic, and SA, under anaerobic conditions (Puziss & Rittenberg, 1957). The marked pH dependence around 5.5 suggested that among these acids, the SA in its partially protonated state (pK_a_'s 4.19 and 5.57) most likely contributed to the effect of Sups. Analysis of Sups generated by several *B. anthracis* strains, including Sterne 34F2 and dSterne, confirmed that SA was present at a concentration of about 1.6 ± 0.2 mM (mean ± SD) (Fig. S4). Supplementation of the culture medium with SA showed that it was toxic to HSAECs in a concentration-dependent manner (shown in Fig. 3b, black bars), but less effective than Sups (Fig. 1a), thus suggesting that Sups contained additional pathogenic factor(s).

**Fig. 4:**
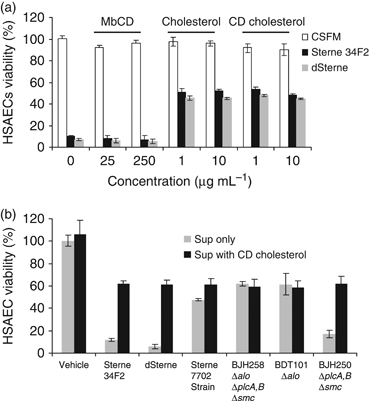
Sups of attenuated and mutant *Bacillus anthracis* strains demonstrate different levels of cholesterol-dependent toxicity. (a) Sups of the Sterne 34F2 and dSterne strains were preincubated with the indicated concentrations of cholesterol or CD-cholesterol for 30 min and tested for toxicity. MbCD served as a control to demonstrate the absence of its effect on the activity of CD-cholesterol. The *X* axis indicates concentrations of added cholesterol, CD-cholesterol, and MbCD. (b) Sups of the indicated *B. anthracis* strains were preincubated with CD-cholesterol (3 μg mL^−1^) for 30 min and tested for toxicity with Alamar Blue as described in Materials and methods. Error bars represent 95% confidence interval of mean.

Experiments with a broad-spectrum protease inhibitor cocktail against aspartic, cysteine, serine, metalloproteases, and aminopeptidases did not reveal any influence of inhibitors on Sup toxicity and therefore argued against the proteolytic cells' damage (Fig. S5). On the other hand, quick permeabilization of the Sup-treated cells evident from the LDH and Trypan Blue tests (Fig. 1a and b) strongly suggested that toxicity was associated with the pH-dependent activity of membrane-damaging secreted bacterial factor. Anthrax hemolysins are potent membrane-damaging proteins and their expression is upregulated under anaerobic conditions (Klichko *et al.*, 2003). ALO is a major *B. anthracis* hemolysin, which belongs to the family of cholesterol-dependent toxins (Tweeten, 2005). We tested the activity of recombinant ALO and found that it caused the formation of membrane blebs similar to the effect of Sups in HSAECs and HUVECs (Movie S1). Damage to membranes of different cell types by PFTs similar to ALO is known to be inhibited by cholesterol (Tweeten, 2005). Recent publications also used a soluble complex of cholesterol with MbCD (CD-cholesterol) (McCormick *et al.*, 2009). In agreement with this, CD-cholesterol protected the cells from membrane blebbing (Fig. 1c). Both cholesterol and CD-cholesterol strongly reduced the toxicity of Sups in the Alamar Blue viability test with HSAECs (Fig. 4a). However, a considerable fraction of toxicity remained cholesterol-independent.

### Deletion of ALO gene abrogates the cholesterol-dependent toxicity

In order to further understand the nature of cholesterol-dependent toxicity, we compared Sups of six *B. anthracis* strains. These strains were comprised of Sterne 34F2, dSterne 34F2, Sterne 7702, and three isogenic mutants of the parental strain Sterne 7702, namely: a single mutant with the deletion of the ALO gene (BDT101); a triple mutant with the deletion of phospholipase A, B, and sphingomyelinase A genes (BJH250); and a quadruple mutant with the deletion of all four hemolysin genes (BJH258). All strains showed similar levels of growth (A^600^) in static 24-h cultures (mean 0.49 ± 0.05 SD) as well as the final pH values of Sups (mean 5.39 ± 0.10 SD). In the presence of cholesterol, the levels of toxicity were also close to each other (mean 39 ± 2% SD), while the cholesterol-dependent toxicity of Sups was variable from strain to strain (Fig. 4b). These results indicated that the cholesterol-independent effect originated from the properties shared by all the tested strains. Because all the deletion mutants and the parental strain 7702 showed the same level of toxicity in the presence of cholesterol, it seemed likely that the cholesterol-independent hemolysins (phospholipases and sphingomyelinase) did not contribute to the toxicity.

As expected, the ALO gene deletion strain BDT101 demonstrated no cholesterol-dependent toxicity (Fig. 4b). The triple mutant BJH250 expressing ALO, but not other hemolysins, showed increased cholesterol dependence in comparison with the parent Sterne 7702. This effect is consistent with the previous data indicating that in this strain, the increased expression of ALO compensates for the absence of other hemolysins (Heffernan *et al.*, 2007). Further deletion of the ALO gene resulting in the quadruple mutant strain, BJH258, abrogated the cholesterol dependence of BJH250. We therefore concluded that the cholesterol-dependent toxicity of Sups was due to the activity of ALO.

To substantiate the above conclusion, we wanted to demonstrate using immunoassays that the cholesterol-binding factor was indeed the ALO. For this purpose, we used a highly sensitive assay developed for the detection of cholesterol-dependent cytolysins (Farrand *et al.*, 2010). ALO from 10 mL of Sups was absorbed to cholesterol spotted onto the PVDF membrane. The membrane with cholesterol-bound ALO was incubated with the rabbit antibody against streptolysin O, which recognizes the highly homologous ALO (Heffernan *et al.*, 2007). The primary antibody was followed by the anti-rabbit goat antibody and the chemiluminescent detection. The results (Fig. S6) are consistent with the cholesterol protection data (Fig. 4b). No ALO signal was detected in the case of ALO deletion mutants, BDT101 and BJH258, while the mutant BJH250 with the deletion of phospholipases and sphingolmyelinase demonstrated increased ALO expression, compared with the parental Sterne 7702. Among the tested strains, dSterne showed the highest amount of ALO.

### ALO and SA contribute to the toxicity of Sups in a synergistic manner

In the next step, we wanted to test whether the activity of ALO could be enhanced by the acidic fermentation products, including SA. For this purpose, we expressed in *E. coli* and purified the recombinant ALO (Klichko *et al.*, 2003). A spike of this protein into Sups strongly increased their activity (Fig. 3a), while at the same concentration, the recombinant ALO was not acutely toxic in the unconditioned CSFM either at pH 7.5 or at 5.3 (Fig. 3b). However, its effect on HSAECs was synergistic with SA at pH 5.3 (Fig. 3b), suggesting that SA could enhance the toxicity of ALO in Sups. Cholesterol completely inhibited the contribution of recombinant ALO to toxicity in the presence of SA, similar to its effect on Sups (Fig. S7).

Control experiments with Sups and ALO in the presence of externally added polymyxin or lipopolysaccharide demonstrated no influence of these compounds on toxicity in a wide range of concentrations (Figs S1 and S8) and therefore argued against the potential interference of lipopolysaccharide contamination with our data.

### Toxic Sups generate ROS causing mitochondrial damage

**Fig. 5:**
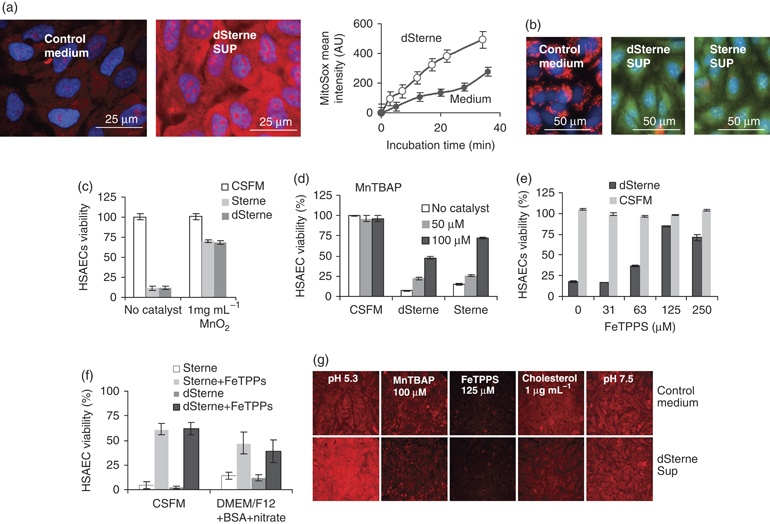
Catalysts of ROS and peroxynitrite decomposition protect HSAECs against mitochondrial damage by Sups. (a) HSAECs grown on coverslips were treated for 30 min with Sups supplemented with 1 μM MitoSox (red) and 2.5 μg mL^−1^ Hoechst 33342 nuclear stain (blue). The cells were washed with HBSS, and the fluorescent images for control medium (left panel) and dSterne Sup-treated cells (middle panel) were recorded. The right panel shows a time course of MitoSox mean fluorescence intensity. Similar results were obtained with Sterne 34F2 Sup (not shown). (b) Cells were washed with HBSS and incubated for 30 min at 37°C, 5% CO_2_ with control CSFM (left panel), dSterne Sup (middle panel), and Sterne 34F2 Sup (right panel). After washing, the cells were stained with JC-1 (10 μg mL^−1^) and Hoechst 33342 (2.5 μg mL^−1^) dyes for 30 min. A dissipation of the mitochondrial potential is evident as a loss of red fluorescence characteristic of aggregated JC-1. (c–f) The Sups generated in CSFM (c–e) and DMEM/F-12 supplemented with 300 μM NaNO_3_ and 1 mg mL^−1^ BSA (f) were mixed with the indicated catalysts for 30 min and used to treat cells for 2 h. (g) Cells were preincubated with catalysts for 30 min, treated with dSterne Sup (bottom row) or control media (top row) for 30 min, and the MitoSox images were recorded as in (a). Where indicated, CD-cholesterol (1 μg mL^−1^) was added to the Sup and control medium for 30 min before incubation with the cells. Additional controls included CSFM titrated with HCl to pH 5.3 (left top panel) and the dSterne Sup titrated to pH 7.5 with NaOH (right bottom panel). Error bars represent 95% confidence interval of mean. A quantitative evaluation of MitoSox fluorescence is presented in Fig. S10.

SA is a substrate of succinate dehydrogenase (or Complex II of the mitochondrial electron transport chain), which converts succinate to fumarate. Under some pathophysiological conditions such as hypoxia, the SA-supported mitochondrial respiration could lead to the formation of damaging ROS (Schonfeld & Wojtczak, 2008). Taking into account that the Alamar Blue dye used in our cell viability test largely reflects the redox activity of the mitochondria, we hypothesized that the mitochondria were the likely targets of the Sup-induced respiratory burst, resulting in the release of ROS and consequent self-intoxication. In agreement with this suggestion, a conventional specific inhibitor of Complex II, thenoyltrifluoroacetone, demonstrated protection of cells from the toxicity of Sups (Fig. 3c). Using MitoSox Red, a peroxide-sensitive probe designed for the detection of mitochondrial ROS production (Fauconnier *et al.*, 2007), we observed a faster accumulation of the dye fluorescence in the presence of Sups, compared with the cells incubated in CSFM at pH 5.3 (Fig. 5a). This finding was further supported in the experiments with dehydrorhodamine 123-loaded HSAECs. The cells treated with Sups showed increased oxidation of this dye to the fluorescent rhodamine in the cytoplasm compared with the pH 5.3-titrated CSFM (not shown). Cell death was accompanied by the quick dissipation of the mitochondrial membrane potential detected with the specific dye, JC-1 (Fig. 5b). This observation is consistent with the mitochondrial damage by ROS (Pacher *et al.*, 2007; Schonfeld & Wojtczak, 2008;) and the reduced ATP content of treated cells (Fig. 1a).

Along with the mitochondria, the plasma membrane NADPH oxidases are often involved in the generation of ROS; however, their contribution to the effect of Sups is likely insignificant, because the inhibitor of these enzymes, diphenyleneiodonium at 10 μM, did not protect the HSAECs from death (not shown). Consistent with ROS formation and their spontaneous or catalytic dismutation to H_2_O_2_, the cells incubated with a well-known catalyst of H_2_O_2_ decomposition, MnO_2_, were partially protected from death in the presence of Sups (Fig. 5c). Control experiments showed that MnO_2_ completely protected the cells from a high concentration (400 mM) of exogenous H_2_O_2_ (Fig. S9). A cell-permeable chemical mimetic of superoxide dismutase, manganese (III) tetrakis (4-benzoyl) porphyrin chloride (MnTBAP), also showed protection in a concentration-dependent manner (Fig. 5d).

We also tested the activity of the metalloporphyrin, 5,10,15,20-tetrakis (4-sulfonatophenyl) porphyrinato iron (III) chloride (FeTPPS). This compound is known to protect cell from the powerful oxidant peroxynitrite formed in the reaction of superoxide anion or H_2_O_2_ at sites of tissue inflammation (Misko *et al.*, 1998; Levrand *et al.*, 2007; Klassen & Rabkin, 2008;). Figure 5 parts e and f show that FeTPPS substantially protected HSAECs from death during the 2-h exposure to Sups from cultures grown in CSFM or DMEM/F-12 medium supplemented with nitrate/BSA. However, these tested catalysts conferred only a temporary protection (the treated cells died within 16 h, data not shown) as it would be expected under the conditions where cell permeabilization by ALO and the effects of low pH were not prevented. In order to confirm that the catalyst treatments indeed caused the decomposition of radical species generated by mitochondria, the fluorescent images of Sup-treated and untreated cells were recorded in the presence or absence of MnTBAP and FeTPPS. Both catalysts reduced MitoSox fluorescence to the level of the corresponding control cells (Figs 5g and S10). CD-cholesterol-treated Sups did not induce MitoSox fluorescence, indicating that the generation of ROS was dependent on the activity of ALO.

## Discussion

In this study, the secreted products of *B. anthracis* generated under microaerobic growth conditions were found to be acutely toxic to HSAECs, HUVECs, Caco-2, and Hep-G2 cells within a few hours. The toxicity of Sups did not correlate with the presence of the toxigenic plasmid XO1 in the tested strains, in agreement with the high resistance of epithelial and endothelial cells to LeTx and EdTx. For example, Paddle *et al.* (2006) reported that LeTx killed HSAECs and HUVECs in a slow process that required at least 3 days. On the other hand, epithelial cells are generally considered sensitive to PFTs (Ratner *et al.*, 2006), and our data demonstrate that ALO can be an important contributor to the toxicity of Sups. The cholesterol-sensitive contribution of ALO is variable between *B. anthracis* strains and is absent in the case of ALO gene knockout mutants. Assuming equal cholesterol-binding affinities of recombinant and native ALO, we estimated that the levels of ALO in Sups were in the range between 70 and 170 pg mL^−1^ (Fig. S6). This estimate indicates that ALO is a highly potent cytotoxin: however, its role in anthrax virulence remains to be elucidated.

A cholesterol-independent toxic effect of Sups is likely relevant to the presence of acidic fermentation products. We show that high toxicity of Sups takes place under the conditions of static culture in the presence of nitrate, forcing the microorganism to switch on the microaerobic acid fermentation, which has not been demonstrated previously for *B. anthracis*. However, gram-positive bacilli are known to produce acidic metabolic products in the microaerobic or anaerobic environment (Puziss & Rittenberg, 1957; Rosenfeld *et al.*, 2005;). This characteristic of *B. anthracis* metabolism seems to be highly relevant to the pathogenicity of the bacterium, because it allows the expression of anaerobic virulence factors under a broad variety of environmental conditions. Although the exact composition of Sups with regard to pathogenic factors in their content remains to be further elucidated, we demonstrate that SA is a toxic fermentation product, which is present in Sups at a millimolar concentration and synergistically enhances the toxicity of recombinant ALO. Because recombinant proteins are typically less active in comparison with the native ones, the potency of ALO in Sups is likely to be higher than it could be inferred from Fig. 3b.

By virtue of the fact that SA serves as an important substrate of the mitochondrial succinate dehydrogenase in the ALO-permeabilized cells, bacterial metabolism can potentially interfere with the host cell respiration through the generation of ROS. Our data are consistent with a mechanism in which the host cells permeabilized by ALO under pathophysiological conditions of increased acidity in the presence of SA intoxicate themselves through the generation of ROS and their secondary products such as H_2_O_2_. Such a mechanism has not been described previously for the bacterial PFTs, although pneumolysin and H_2_O_2_ were reported to be synergistically toxic to the lung epithelium (Feldman *et al.*, 2002).

While the mechanistic details of Sup-induced cells death require further studies, it is likely that the membrane pores formed by ALO facilitate the SA access to the cell cytoplasm. SA is known as a pathogenic factor of *Bacteroids* species inhibiting neutrophils at pH 5.5, but not at pH 7 (Rotstein *et al.*, 1987). Many classical PFTs create membrane channels, which are highly permeable to small molecules such as SA (Walev *et al.*, 2001; Tilley *et al.*, 2005;). Acidification of the cell cytoplasm and the intermembrane mitochondrial space might result in the high electrochemical gradient across the inner membrane known to inhibit electron transport or even cause a reverse flow of electrons in the respiratory chain (Lambert & Brand, 2004). In this case, the mitochondria are expected to generate increased amounts of ROS. It is therefore plausible that such a mechanism could be broadly relevant to a number of infectious agents capable of PFT expression and acid fermentation under microaerobic or strict anaerobic conditions.

The fate of ROS generated under pathophysiological conditions of infectious process is complex. Peroxide radical and the product of its dismutation, H_2_O_2_, can generate secondary species with higher toxicity such as peroxynitrite in reaction with nitric oxide and might thus enhance the synergistic effect of ALO and SA. Nitric oxide is often present in a biological environment (Zweier *et al.*, 1999). It can also be released by denitrifying bacteria and trapped in the reactive, but nontoxic form by serum albumin (Pettersen *et al.*, 2005; Rock *et al.*, 2005;). The toxicity of peroxynitrite has been implicated in a number of biological processes; however, fast reactions of peroxynitrite make it difficult to identify its formation unambiguously. A catalytic decomposition of peroxynitrite by superoxide dismutase and its mimics (e.g. metalloporphyrins) is also often used to indirectly confirm the peroxynitrite presence. Our experiments with dehydrorhodamine 123, MnTBAP, and FeTPPS support this possibility, because it has been found recently that these compounds represent true scavengers of peroxynitrite rather than ROS (Glebska & Koppenol, 2003; Pacher *et al.*, 2007;). In agreement with our suggestion, experiments with monkeys intravenously challenged with vegetative Sterne 34F2 bacteria also demonstrated a strong pattern of lung tissue nitration indicative of the peroxynitrite formation (Stearns-Kurosawa *et al.*, 2006).

The findings we report are limited to *in vitro* observations with attenuated *B. anthracis* strains and need to be substantiated in further experiments with virulent strains. It has been shown recently that neither LeTx nor EdTx are required for the high virulence of Ames and UT500 strains in mice (Heninger *et al.*, 2006; Chand *et al.*, 2009;). It is intriguing to test whether the mechanism we suggest is operable with these strains under conditions of systemic hypoxia and global acidosis invariably found in terminal anthrax patients and moribund experimental animals. However, the contribution of *B. anthracis* to metabolic acidosis, a life-threatening pathogenic condition affecting cardiac, vascular, and epithelial functions, has received little attention in anthrax research. Although metabolic acidosis driven by both the bacteria and the host can itself be a factor inducing increased barrier permeability and the release of ROS, the pH-dependent action of SA and ALO might further contribute to anthrax pathogenicity.

Pertinent to our observations, it has been shown that *Pseudomonas aeruginosa* grows in the mucus on the lung surface of cystic fibrosis patients under the microaerobic conditions resembling those of static cultures (Worlitzsch *et al.*, 2002). The growth is supported by nitrate contained in the airway surface fluid at concentration as high as 400 μM. In response to the hypoxic environment, the destructive lung capacity of bacterium is increased (Worlitzsch *et al.*, 2002). However, the biological relevance of our findings might not be limited to the lung epithelium. ALO is broadly toxic to different cell types and therefore organs or specific locations such as thrombotic vessels deprived of oxygen during the disease progression are expected to be the likely sites for the generation of the ALO-SA toxicity by a large number of bacteria found in the circulation and the thrombi. It needs to be emphasized that even in a physiological norm, the lower oxygen tension in many host tissues, compared with the aerated bacterial culture, might stimulate microaerobic fermentation. ALO has been shown to damage lung circulation through the formation of thrombi (Cowan *et al.*, 2007) and increase the barrier permeability of lung and gut epithelial cells (Popova *et al.*, 2006; Bishop *et al.*, 2010;). The intracellular pH in the ischemic tissue can decline within minutes to a value as low as 5.5 (Zweier *et al.*, 1999). One might expect that breaching the protective barriers of vasculature and epithelia by the combined action of ALO, LeTx and EdTx, other proteases, and hemolysins may further reduce oxygenation and set up a self-perpetuating pathological circle.

In summary, our *in vitro* data suggest a new virulence mechanism when metabolic acidosis, hypoxia, and the pore-forming protein generate a toxic milieu killing the cell. Studies aimed to test the relevance of this mechanism to anthrax pathogenicity *in vivo* are currently in progress.

## Statement

Re-use of this article is permitted in accordance with the Terms and Conditions set out at: http://wileyonlinelibrary.com/onlineopen#OnlineOpen_Terms

## SupportingInformation

**Fig. S1.** Lipopolysaccharide does not demonstrate toxicity to HSAECs at concentration up to 10 ngmL^−1^ upon incubation with cells for 2 h at 37°C, 5% CO_2_ in CSFM (pH 7.5) or in the presence of 10mM SA in CSFM titrated to pH 5.4 with HCl.

**Fig. S2.** Calibration curves of the SA assay obtained with SA dissolved in water (closed circles), CSFM (open circles) or spiked into Sterne 34F2 Sup (diamonds).

**Fig. S3.** Toxicity of Sups is concentration-dependent.

**Fig. S4.** The SA content of static *Bacillus anthracis* cultures of indicated strains grown in CSFM for 24 h.

**Fig. S5.** Broad-spectrum protease inhibitor cocktail against aspartic, cysteine, serine, metalloproteases and aminopeptidases (1 × Halt cocktail, Pierce, supplemented with 1mM phenanthroline and 200 μM phosphoramidon) does not protect HSAECs from Sup toxicity.

**Fig. S6.** Dot-blot assay of the ALO in Sups.

**Fig. S7.** Cholesterol and water-soluble cholesterol (CDcholesterol) inhibit toxicity of ALO in the presence of SA.

**Fig. S8.** Polymyxin does not influence the toxicity of Sups (a) and ALO in combination with SA (b) to HSAECs.

**Fig. S9.** MnO_2_ protects of HSAECS from externally added H_2_O_2_.

**Fig. S10.** Quantitation of results presented in Fig. S5g.

**Movie S1.** Recombinant ALO causes HUVECs' cytoplasmic membrane blebbing.
