# Supplementary figures and images for: Anthrolysin O and fermentation products mediate the toxicity of Bacillus anthracis to lung epithelial cells under microaerobic conditions
Source: FEMS Immunol Med Microbiol. 2010 Jan 14;61(1):15–27. doi: 10.1111/j.1574-695X.2010.00740.x (PMC3040846; doi:10.1111/j.1574-695X.2010.00740.x)

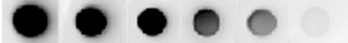

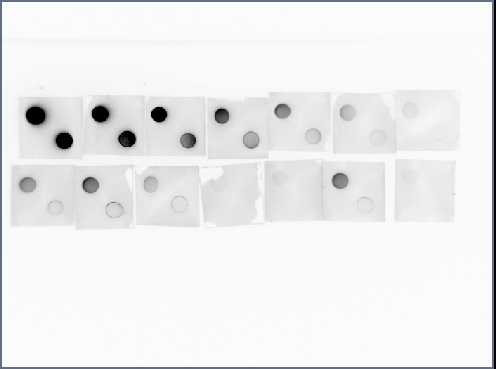


2500 1000 500 250 100 50 0 pg/ml of ALO

CSFM Sterne dSterne Sterne BJH BDT BJH

34F2 7702 258 101 250

**a**

**b**

**c**

Popova et al. Supplemental Fig. 6

Supplement: Supplementary file 6 [file fim0061-0015-SD6.doc]
