# Supplementary material for: Anthrolysin O and fermentation products mediate the toxicity of Bacillus anthracis to lung epithelial cells under microaerobic conditions
Source: FEMS Immunol Med Microbiol. 2010 Jan 14;61(1):15–27. doi: 10.1111/j.1574-695X.2010.00740.x (PMC3040846; doi:10.1111/j.1574-695X.2010.00740.x)
Supplement: Supplementary file 7 [file fim0061-0015-SD7.doc]

CD-cholesterol

MbCD

Cholesterol

Concentration (µg/ml)

Popova et al. Supplemental Fig. 7
